# HIF‐1α Promotes the Confined Migration of Gastric Cancer Cells by Modulating Phosphatidylcholine Metabolism

**DOI:** 10.1111/jcmm.70828

**Published:** 2025-09-15

**Authors:** Ming Zhou, Kanger Shen, Ziyi Huang, Qin Zhan, Jiayu Wang, Xiaozhe Mao, Tongguo Shi, Rui Li

**Affiliations:** ^1^ Jiangsu Institute of Clinical Immunology The First Affiliated Hospital of Soochow University Suzhou China; ^2^ Jiangsu Key Laboratory of Clinical Immunology Soochow University Suzhou China; ^3^ Department of Gastroenterology The First Affiliated Hospital of Soochow University Suzhou China

**Keywords:** CEPT1, confined migration, gastric cancer, HIF‐1α, phosphatidylcholine

## Abstract

Confined migration (CM) is a key step in the process of cancer cell metastasis. However, the molecular mechanisms underlying CM in gastric cancer (GC) remain unclear. Here, we found that CM‐GC cells had remarkable metastatic and antiapoptotic capacities. Moreover, we observed that HIF‐1α was highly expressed in CM‐GC cells. HIF‐1α knockdown reversed the enhanced metastatic and antiapoptotic capacities of CM‐GC cells in vitro and in vivo. Mechanistically, HIF‐1α enhanced the metastatic and antiapoptotic capacities of CM‐GC cells through phosphatidylcholine (PC) synthesis. Importantly, the addition of L‐α‐phosphatidylcholine (L‐α‐PC), a natural PC, markedly increased the metastatic and antiapoptotic capacities of GC cells, similar to CM GC cells. In addition, we observed that increased HIF‐1α protein expression was positively correlated with the expression of CEPT1, a key enzyme of PC synthesis, in GC tissue samples. Furthermore, high expression of HIF‐1α and CEPT1 in GC tissues predicted poor prognosis in patients with GC. Overall, our study supports the crucial role of HIF‐1α in the CM of GC cells and provides novel therapeutic targets and strategies for metastatic GC.

## 1 Introduction

GC is a common malignancy and one of the leading causes of death from cancer worldwide. According to GLOBOCAN's 2022 data, the incidence of GC accounted for 4.9% of the world's new cancer cases in 2022, whereas the mortality of GC accounted for 6.8%, both of which were fifth in the global ranking [1, 2]. Although the survival rate of patients with GC has been greatly improved by increasing the early detection rate and improving treatment methods, the 5‐year survival rate of patients with GC in China has not shown a significant downwards trend in recent years [3]. The primary factors affecting the prognosis of patients with GC are recurrence and distant metastasis [4]. Therefore, understanding the underlying mechanisms of GC metastasis has great clinical value.

Confined migration (CM) is the migration of normal or tumour cells through confined spaces by changing their shape and size, a process that overcomes the mechanical stress challenges of a restrictive physical environment [5]. CM is closely related to the malignant potential of tumour cells, such as their metastatic and antiapoptotic capacities. For example, CM upregulates antiapoptotic proteins (IAPs) to reduce apoptotic caspase activation in breast cancer cells and increase cell motility and immune escape, thereby promoting tumour metastasis [6]. Recent studies have shown that IGFBP1, which is upregulated in CM cells, inhibits the phosphorylation of superoxide dismutase (SOD2) serine (S) 27 by inhibiting AKT1 activity, ultimately leading to increased metastatic capacity [7]. However, the underlying mechanism of CM in GC remains unclear.

A hypoxic tumour microenvironment is a common feature of solid tumours, including in GC [8]. HIF‐1, a heterodimer consisting of two subunits, HIF1α and HIF1β, is the most important member of the HIF family. Tumours often adapt to low oxygen levels by upregulating HIF‐1α expression through metabolic reprogramming, thereby promoting tumour cell proliferation and metastasis [9, 10]. Under normal oxygen conditions, HIF‐1α is often hydroxylated by HIF hydroxylase for degradation via the ubiquitin‐proteasome pathway. In contrast, HIF‐1α is often stable under hypoxic conditions and is associated with poor tumour prognosis [11]. Numerous studies have shown that HIF‐1α expression in GC tissues is significantly upregulated and closely related to multiple biological functions, including metastasis [12, 13]. For example, HIF‐1α acts as a transcription factor to upregulate ACLY expression in GC cells, resulting in tumorigenesis and peritoneal metastasis of GC. Nevertheless, the role and molecular mechanism of HIF‐1α in the CM of GC cells have rarely been reported.

Here, we successfully constructed a CM GC cell model and found that CM GC cells had enhanced metastatic and antiapoptotic capacities. Moreover, we observed that HIF‐1α was significantly increased in CM GC cells and that depletion of HIF‐1α abolished the enhanced metastatic and antiapoptotic capacities of CM GC cells through CEPT1/PC metabolism. This finding indicates that the prevention of CM of GC cells via blockade of the HIF‐1α/CEPT1/PC pathway may constitute a novel therapeutic strategy for metastatic GC.

## 2 Materials and Methods

### 2.1 Cell Culture

The human GC cell lines AGS and HGC27 (ATCC, Manassas, VA, USA) were cultured in RPMI‐1640 medium (EallBio, Beijing, China, #03.4007‐C) and DMEM (EallBio, #03.100‐6C) at 37°C and 5% CO_2_. All of the above media were supplemented with 10% foetal bovine serum (FBS, EallBio, #3 U16001DC) and 1% penicillin‐streptomycin (NCM Biotech, Suzhou, China, #C100C‐5).

### 2.2 Construction of the CM Cell Model

A chamber (Corning, NY, USA, #3420) with a pore size of 3 μm was placed in a 6‐well plate. A substrate gel (Corning, #356234) prepared with serum‐free medium at a ratio of 1:20 was added to the upper chamber, which was incubated at 37°C for 2 h and hydrated with 100 μL of sterile PBS for 30 min. Next, 1.5 mL of culture medium with 20% FBS was added to the lower chamber, and a 1.5 mL cell suspension (1 × 10^6^ cells) in serum‐free culture medium was added to the upper chamber. After 46 h in the incubator, we aspirated the medium from both the upper and lower parts of the chamber and cleaned the lower part with PBS. Subsequently, the cells in the lower part of the chamber were digested using 1 mL of trypsin (Beyotime, #C0203). After terminating the digestion with 1 mL of medium containing 10% FBS, we transferred the cells from the lower part of the chamber into a six‐well plate by gently blowing. The cells collected were identified as CM cells [14, 15].

### 2.3 Cell Transfection

A commercially available small interfering RNA (siRNA) targeting HIF‐1α was obtained from RiboBio (Guangzhou, China). Si‐Scramble was used as a negative control (NC). Candidate GC cells at a density of 30% were infected with HIF‐1α siRNA using Lipo8000TM transfection reagent (Beyotime, #C0533). After 72 h, the infection efficacy was verified via Western blotting.

### 2.4 Hypoxia‐Induced Environment

Before conducting the apoptosis‐related experiment, 400 μmol of cobalt chloride (MERCK, Germany, #255599‐5G) was added to a petri dish containing cells that had reached 90% confluence. The cells were then cultured in an incubator for 24 h, after which Western blot and apoptosis assays were performed.

### 2.5 Western Blot Analysis

In accordance with the number of cells collected, an appropriate amount of RIPA lysis buffer (Beyotime, #P0013) containing protease and phosphatase inhibitors (Beyotime, #P1045) was added, and the mixture was ultrasonicated on ice for 1 min to ensure full lysis. The concentration of the collected protein supernatants was determined using a BCA protein assay kit (Beyotime, #P0011). A 10% SDS‐PAGE gel rapid preparation kit (NCM Biotech, #P2012) was used to configure the upper and lower layers of the gel and separate 30 μg of protein sample. The proteins were then transferred to a PVDF membrane with a pore size of 0.45 μm preactivated with anhydrous methanol (GE Healthcare Life Sciences, Germany, #10600023). The PVDF membrane was placed in a 5% bovine serum albumin (BSA) solution (Fcmacs, Nanjing, China, #FMS‐WB021) and incubated at room temperature for 1 h. The membrane was cut according to the size of the target protein molecule and incubated with the corresponding primary antibody at 4°C overnight. The primary antibodies utilised included anti‐HIF‐1α rabbit polyclonal antibodies (CST, USA, MA #36169), anti‐CEPT1 rabbit polyclonal antibodies (Proteintech, IL, USA #20496‐1‐AP), anti‐caspase 3 rabbit monoclonal antibodies (Beyotime, Shanghai, China #AC033), anti‐PARP1 rabbit polyclonal antibodies (Proteintech, IL, USA #13371‐1‐AP) and anti‐GAPDH mouse monoclonal antibodies (Proteintech, IL, USA #60004‐1‐Ig). Then, the corresponding secondary antibodies were added and incubated at room temperature for 1 h. Finally, the PVDF membrane was developed using ECL reagent (NCM Biotech, #10,100) and a ChemiDocTM MP imaging system (Bio‐Rad, CA, USA).

### 2.6 Flow Cytometry

The cell samples were collected in the flow tube in advance, washed once with PBS, centrifuged, and then resuspended in 1× binding buffer. In accordance with the instructions of the PE Annexin V apoptosis detection Kit I (BD Biosciences, NJ, USA, #559763), the corresponding Annexin V and 7‐AAD were added to different tubes and incubated at room temperature for 20 min in the dark. Flow cytometry (Beckman Coulter, CA, USA) was used to detect the percentage of apoptotic cells. In this study, Annexin V+, 7‐AAD‐ and Annexin V+, 7‐AAD+ cells were interpreted as apoptotic cells.

### 2.7 Transwell Migration and Invasion Assays

For both the transwell migration and invasion experiments, a Falcon cell culture chamber (Corning, NY, USA, #353097) with an aperture of 8 μm was first placed in a 24‐well plate. Then, 400 μL of 20% serum‐containing medium was added to the lower chamber, and a cell suspension (4 × 10^4^ cells) with 400 μL of serum‐free medium was added to the upper chamber. For the invasion experiment, the bottom of the cell culture chamber was coated with a matrix gel (Corning, #356234) prepared in serum‐free medium at a ratio of 1:30. After culturing for 24–48 h, the migrated or invasive cells were fixed with 4% paraformaldehyde and stained with crystal violet staining solution. Finally, the cells were observed and photographed under a 200× inverted microscope (Nikon, Tokyo, Japan).

### 2.8 Real‐Time Quantitative PCR (RT–qPCR)

Total RNA was extracted from WT and CM cells using an RNA extraction kit (Yishan Biotech, Shanghai, China, #RN001). The expression of HIF‐1α was detected using a reverse transcription system (Monad, #140733) and a qPCR kit (Monad, #220625). Reverse transcription conditions are: at 37°C for 15 min, then at 85°C for 5 s. The conditions for qPCR are set to a 5 min initial denaturation step at 95°C followed by 40 amplification cycles consisting of a 10 s denaturation step at 95°C and a 30 s annealing/extension step at 60°C. The abundance of mRNA was represented by the cycle threshold (CT) cycle method. GAPDH was used to normalise the expression. The primers used in RT–qPCR for the detection of *HIF1‐α* and *GAPDH* were as follows: *HIF1‐α*: Forward: 5′‐GAACGTCGAAAAGAAAAGTCTCG‐3′, Reverse: 5′‐CCTTATCAAGATGCGAACTCACA‐3′; *GAPDH*: Forward: 5′‐GGAGCGAGATCCCTCCAAAAT‐3′, Reverse: 5′‐GGCTGTTGTCATACTTCTCATGG‐3′.

### 2.9 Nontargeted Metabolomics Analysis

Untargeted metabolomics analysis of WT‐HGC27 and CM‐HGC27 cells was performed by Shanghai Weihuan Biotechnology Co. Ltd. (Shanghai, China). Principal component analysis (PCA) was used to examine the multivariate raw data, facilitating the identification of groupings, trends, and outliers among the observed variables within the dataset. The partial least squares discriminant analysis (PLS‐DA) method, with a variable importance in projection (VIP) score of ≥ 1 for the first two principal components of the model, along with fold change (|log2 (fold change)| > 2) and Student's *t* test (*p* < 0.05), was used to identify differentially abundant metabolites.

### 2.10 ELISA

A human phosphatidylcholine ELISA kit (TW‐reagent, Shanghai, China #TW1580705), a human triglyceride ELISA kit (TW‐reagent, #TW1580901) and a human diacylglycerol ELISA kit (TW‐reagent, #TW1580663) were used to measure the levels of phosphatidylcholine, triglyceride and diacylglycerol in the cell supernatant according to the kit instructions.

### 2.11 Chromatin Immunoprecipitation (ChIP) Assay

The ChIP test is performed using the EZ‐ChIP test kit (MERCK, Germany, #17–371) as described in the instructions. In short, AGS and HGC27 cells treated with cobalt chloride were treated with 37% formaldehyde for 10 min to induce chromatin cross‐linking. Next, the chromatin is cut by ultrasound into DNA fragments 100 to 1000 base pairs long. The immunoprecipitation procedure was conducted utilising an anti‐HIF‐1α rabbit anti‐clonal antibody (CST, USA, MA #A700‐001). As a negative control, normal rabbit IgG was employed. Then, the complex was eluted, de‐crosslinked and purified. Purified chromatin was amplified by PCR using three primers specifically created for the CEPT1 promoter region, namely P1 (Forward: 5′‐CAACAGGAACGTGTACTCAACAGG‐3′, Reverse: 5′‐ATTAATTCCTCTGGTTTGTTGAAGC‐3′), P2 (Forward: 5′‐TGATCAGTATGTACTTGTTCCTCTT‐3′, Reverse: 5′‐AAGGTGCAGGTATGAGCAGG‐3′) and P3 (Forward: 5′‐CCAACATGGTGACACCCTGT‐3′, Reverse: 5′‐TCCCGCGTTCAAGTGATTCT‐3′). Finally, the obtained DNA fragments were analysed using agarose gel electrophoresis.

### 2.12 GC Tissue Microarray and Immunohistochemistry (IHC)

A GC tissue microarray chip (#HStmA180Su19) including 94 GC tissue samples and 86 adjacent normal tissue samples was purchased from Shanghai Outdo Biotech Co. Ltd. (Shanghai, China). For the IHC assay, a GC tissue microarray chip and paraffin‐embedded mouse lung tissue were incubated with an anti‐HIF‐1α rabbit polyclonal antibody (CST, USA, MA #36169) and an anti‐CEPT1 rabbit polyclonal antibody (Proteintech, IL, USA #20496‐1‐AP) in a humidified refrigerator overnight at 4°C. The tissue was then covered with the diluent of HRP‐conjugated goat anti‐rabbit IgG at 37°C for 30 min, followed by incubation with 3,3′‐diaminobenzidine (DAB). Two experienced pathologists determined the IHC score. The IHC scoring criteria were as described previously. Final scores were calculated by multiplying the intensity of staining (negative: 0; mild: 1; moderate: 2; severe: 3) by the area of staining (1; ≤ 25%: 2; > 25% and ≤ 50%: 3; > 50% and ≤ 75%: 4; > 75%). The scores ranged from 0 to 12. The Medical Ethics Committee of the First Affiliated Hospital of Soochow University (Suzhou, China) approved the study protocol (Ethics No. 2024541). Informed consent was obtained from each patient.

### 2.13 Murine Lung Metastasis Model

Nine 6‐week‐old female NSG mice purchased from Shanghai Laboratory Animal Center (Shanghai, China) were randomly divided into three groups: the WT‐HGC27 group, CM‐HGC27 group and WT‐HGC27 + L‐α‐PC group. The mice in the HGC27 group and HGC27 + L‐α‐PC group were injected with 100 μL of a PBS suspension containing 2 × 10^6^ HGC27 cells through the tail vein, and those in the CM‐HGC27 group were injected with the same number of CM‐HGC27 cells. For the WT‐HGC27 + L‐alpha‐PC group, the mice were injected intraperitoneally weekly with 100 μL of L‐alpha‐PC (10 mg/kg, Sigma–Aldrich, MO, USA; # P3556) dissolved in PBS, and the remaining two groups were injected with the same volume of PBS. After 8 weeks, the mice were sacrificed, and their lung tissues were removed, immersed in 4% paraformaldehyde and embedded in paraffin. The paraffin‐embedded mouse lung tissue was used for subsequent haematoxylin‐eosin (HE) and IHC experiments.

### 2.14 HE Staining

The fixed mouse lung tissues were dehydrated, embedded successively, and then cut into 5 μm tissue sections. The sections were then stained with a haematoxylin and eosin staining kit (Beyotime, #C0105) according to the manufacturer's instructions.

### 2.15 Statistical Analysis

GraphPad Prism (9.0) was used to statistically analyse the data and generate bar graphs. FlowJo (10.8.1) software was used to analyse the flow cytometry data. ImageJ software was used to analyse the grey values of the Western blot images. Student's *t* test and one‐way ANOVA were used for statistical significance. Pearson correlation analysis was used to study the relationship between HIF‐1α and CEPT1. The Kaplan–Meier method with the log‐rank test was used for survival analysis. The data are expressed as the means ± SDs or SEMs. *p < 0.05, **p < 0.01 and ***p < 0.001 were considered statistically significant.

## 3 Results

### 3.1 CM GC Cells Have Increased Metastatic and Antiapoptotic Capacities

**FIGURE 1:**
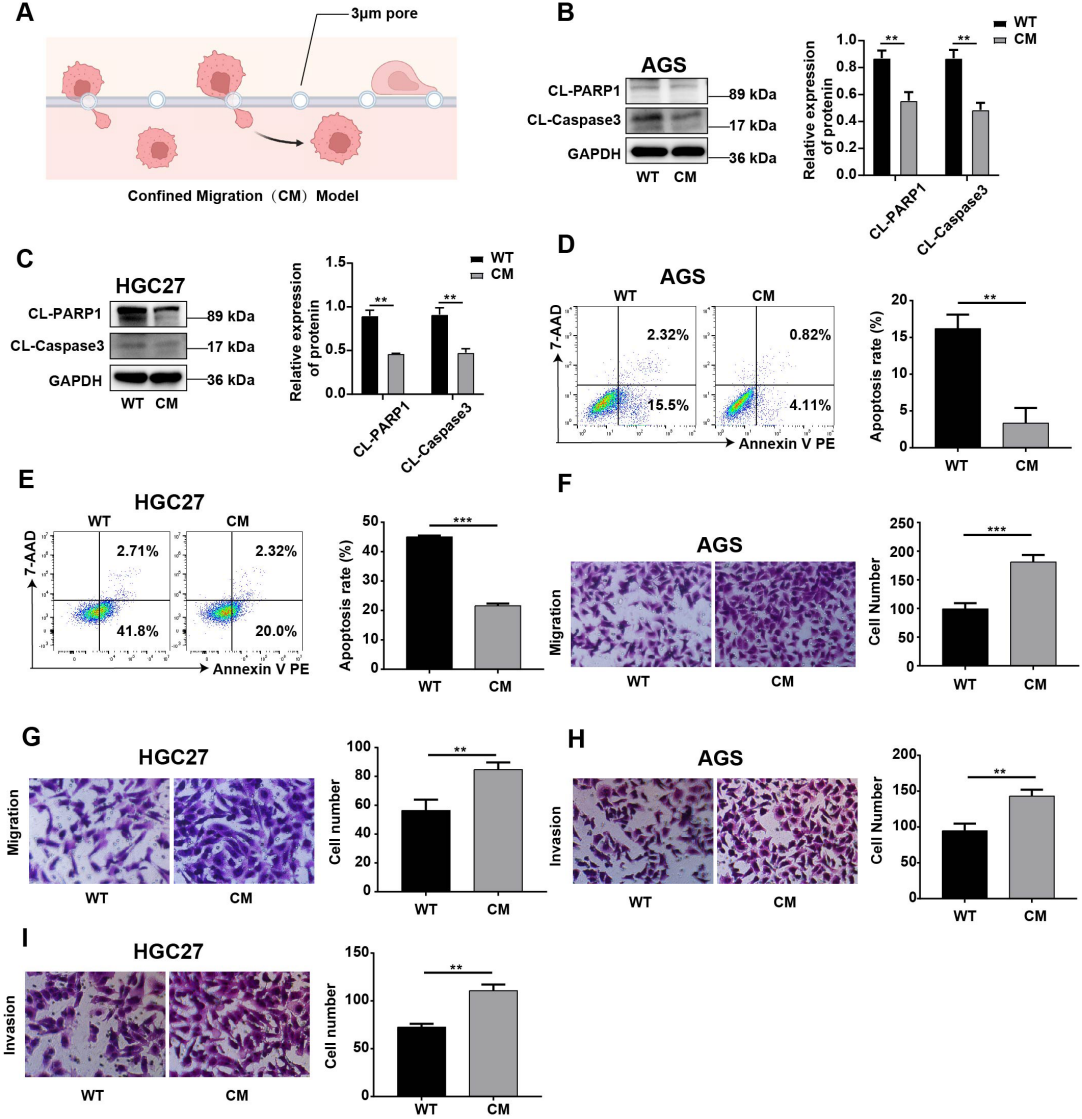
CM‐GC cells showed increased metastatic and antiapoptotic capacities. (A) Two gastric cancer cell lines, AGS and HGC‐27, were passed through a 3 μm diameter chamber for 46 h in the incubator to construct a CM‐GC cell model. (B, C) Western blot analysis was used to assess the changes in CL‐PARP1 and CL‐Caspase3 protein levels in AGS and HGC27 treated with CM. GAPDH served as a loading control. (D, E) The apoptosis rates of AGS and HGC27 after CM treatment were detected by flow cytometry. (F–I) Transwell assays were used to detect the migration and invasion of AGS and HGC27 after CM treatment. The experiment was performed in three separate sessions. The values are expressed as the standard deviation (SD), and Student's *t* test was used to assess statistical significance. Nonsignificant results are represented by ‘ns’, and significant levels are represented by *p < 0.05, **p < 0.01 and ***p < 0.001.

To investigate the effects of CM on GC cells, we utilised AGS and HGC27 cells, both of which possess certain metastatic potential [14, 15], to construct CM models of GC cells. Specifically, AGS and HGC27 cells were passed through a chamber with a diameter of 3 μm via a serum gradient (Figure 1A). Compared with those in WT cells, the protein expression levels of CL‐Caspase3 and CL‐PARP1 were significantly lower in CM‐AGS and CM‐HGC27 cells under hypoxic conditions (Figure 1B,C). Flow cytometry confirmed the reduced apoptosis rate of CM cells under hypoxic conditions (Figure 1D,E). In addition, compared with those of WT cells, the migration and invasion capacities of CM cells were considerably improved (Figure 1F–I). In summary, these results show that CM leads to an increase in the metastatic and antiapoptotic capacities of GC cells.

### 3.2 HIF‐1α Is Associated With the Antiapoptotic and Metastatic Characteristics of CM GC Cells

**FIGURE 2:**
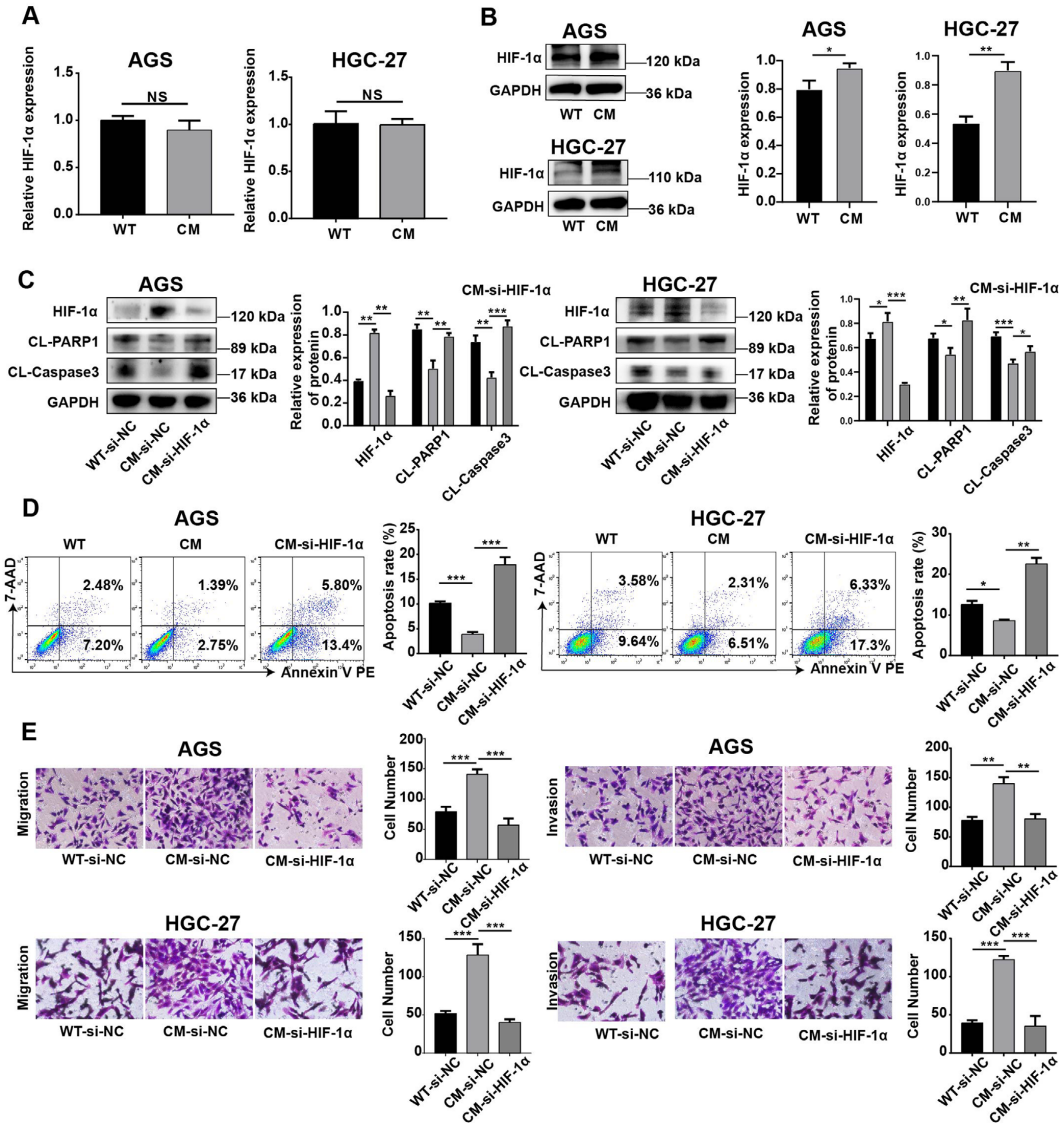
HIF‐1α is involved in the metastatic and antiapoptotic capacities of CM‐GC cells. (A) The expression of the HIF‐1α gene in AGS and HGC27 treated with CM was detected by RT–qPCR. (B) Western blot analysis of the changes in HIF‐1α protein levels in AGS and HGC27 after CM treatment. (C) The protein expression of HIF‐1α, CL‐PARP1 and CL‐Caspase3 in HIF‐1α‐knockdown CM from GC cells. (D) The apoptosis rates of CM‐AGS and CM‐HGC27 with HIF‐1α knockdown were detected by flow cytometry. (E) Transwell assays were used to detect the migration and invasion of CM‐AGS and CM‐HGC27 after knocking down HIF‐1α. The experiment was performed in three separate sessions. The values are expressed as the standard deviation (SD), and Student's t test was used to assess statistical significance. Nonsignificant results are represented by ‘ns’, and significant levels are represented by *p < 0.05, **p < 0.01 and ***p < 0.001.

Multiple previous studies have shown that HIF‐1α is involved in the regulation of many important steps of the metastatic processes of cancers [16]. Therefore, we next asked whether HIF‐1α participates in CM processes of GC cells. To this end, we detected *HIF‐1α* mRNA and protein expression in CM GC cells under hypoxic conditions via RT–qPCR and Western blotting. Although there was no statistically significant difference in the expression of *HIF‐1α* mRNA between CM cells and WT cells (Figure 2A), the HIF‐1α protein levels were significantly increased in CM cells (Figure 2B). To investigate the role of high HIF‐1α expression in regulating antihypoxia and metastasis in CM cells, a commercial HIF‐1α siRNA was used, which resulted in a significant decrease in HIF‐1α protein expression (Figure 2C). Silencing of HIF‐1α counteracted the effects of CM on CL‐PARP1 and CL‐Caspase3 expression in GC cells as well as the apoptosis rate (Figure 2C,D). Furthermore, the transwell results also revealed that the enhanced migration and invasion capacity of GC cells induced by CM was abolished by the knockdown of HIF‐1α (Figure 2E). These results show that the metastatic and antiapoptotic capacities of CM GC cells are associated with HIF‐1α.

### 3.3 HIF‐1α Promotes the Antiapoptotic and Metastatic Characteristics of CM GC Cells via PC Metabolism

**FIGURE 3:**
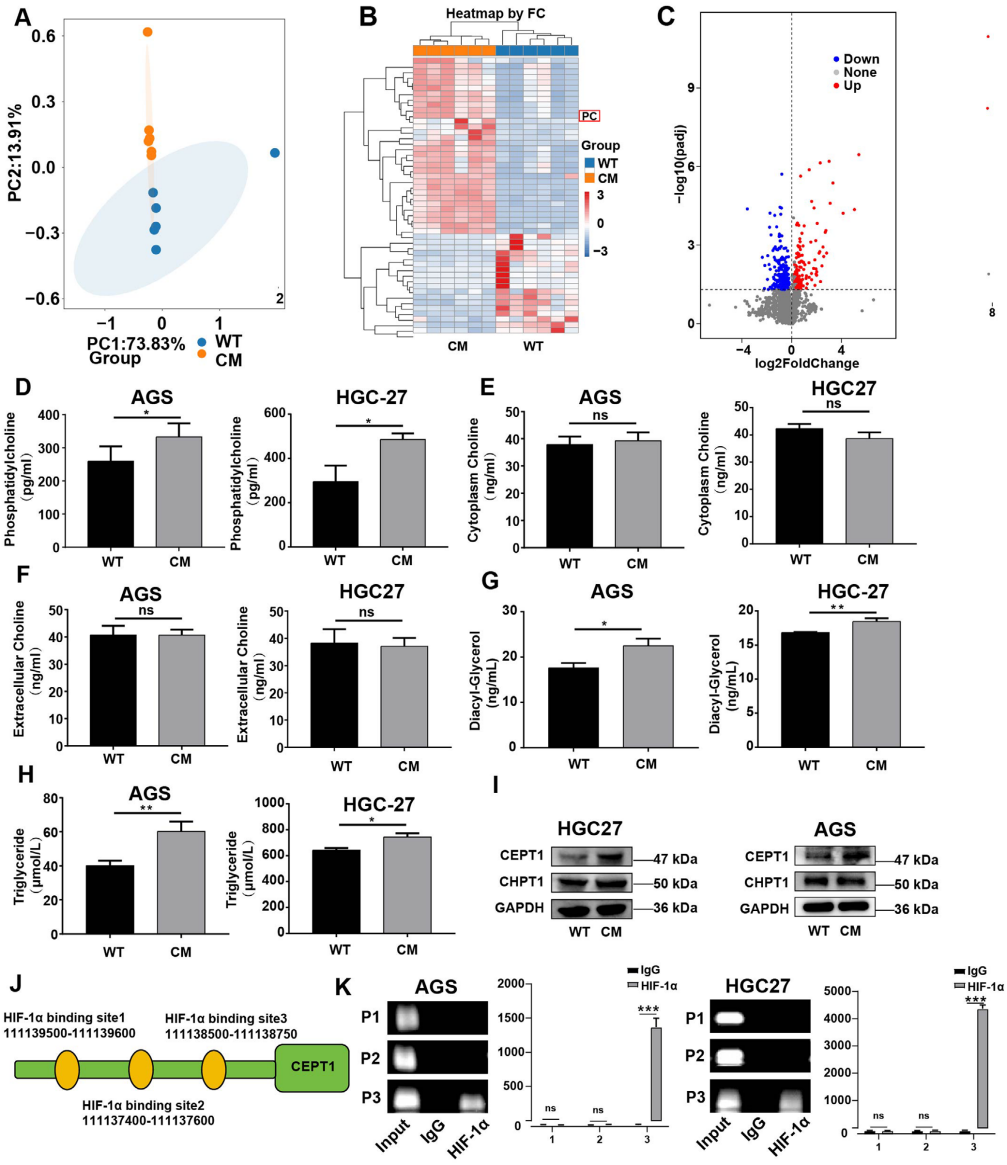
CEPT1 is involved in the regulation of PC synthesis in CM from gastric cancer cells. (A) PCA of nontargeted metabolomics data from gastric cancer cells in the WT‐HGC27 and CM‐HGC27 groups. (B, C) Volcano map of differentially abundant metabolites in WT‐HGC27 and CM‐HGC27 GC cells. (D) PC content in the supernatants of CM‐AGS and CM‐HGC27. (E, F) Choline content in the cell supernatants and intracellular fractions of CM‐AGS and CM‐HGC27. (G) Changes in the diacylglycerol content in the supernatants of CM‐AGS and CM‐HGC27. (H) Changes in the triglyceride content in the supernatants of CM‐AGS and CM‐HGC27. (I) Protein levels of CHPT1 and CEPT1 in CM‐AGS and CM‐HGC27. (J) The schematic diagram shows that there are three HIF‐1α binding sites in the 5′ region of the CEPT1 promoter. (K) ChIP analysis was performed to evaluate the binding of HIF‐1α to the CEPT1 promoter in GC cells. Normal rabbit IgG was used as control. The values are expressed as the mean values (SEM). Five samples were analysed for each condition, and the experiment was conducted three times. *p < 0.05.

To investigate the potential mechanism of the effect of HIF‐1α on the metastatic and antiapoptotic capacities of CM GC cells, we performed nontargeted metabolomics analysis using WT and CM GC cells. PCA revealed variations in the levels of metabolites between WT and CM GC cells (Figure 3A). As shown in Figure 3B and Table S1, 371 differentially expressed metabolites, specifically, 226 downregulated metabolites and 145 upregulated metabolites (|log2 (fold change)| > 2 and *p* < 0.05), were identified in CM‐HGC27 cells. Among these differentially expressed metabolites, phosphatidylcholine (PC) was significantly upregulated in CM‐HGC27 cells (Figure 3C). Given that PC can modulate cancer cell survival and metastasis [17, 18], we hypothesised that HIF‐1α enhances the metastatic and antiapoptotic capacities of CM GC cells via the PC content. In the case of hypoxia, the PC content in the supernatant of CM GC cells was significantly greater than that in the WT group (Figure 3D). Given that the Kennedy pathway and the triglyceride–diacylglycerol–PC pathway are two crucial pathways for PC synthesis [19, 20], we next investigated which pathway is involved in PC synthesis in CM GC cells. As shown in Figure 3E,F, the levels of choline, the raw material of the Kennedy pathway, were not significantly different in either the supernatant or in the cells under hypoxic conditions. In contrast, under hypoxic conditions, the contents of triglycerides and diacylglycerol, two raw materials of the triglyceride–diacylglycerol–PC pathway, were significantly greater in CM cells than in WT cells (Figure 3G,H). Moreover, the Western blot results revealed no significant difference in the expression of CHPT1, a key enzyme of the Kennedy pathway, in CM cells, whereas there was a notable increase in the expression of CEPT1, a crucial enzyme of the triglyceride–diacylglycerol–PC pathway (Figure 3I and Figure S1). To further investigate whether and how HIF‐1α regulates CEPT1 expression in GC cells, we performed ChIP assays. We utilised the JASPAR database to predict the binding site for CEPT1. Subsequently, we identified three HIF‐1α binding sites within the CEPT1 promoter region, namely P1, P2 and P3 (Figure 3J). The ChIP assay results revealed a high concentration of DNA in the P3 region when purified with anti‐HIF‐1α antibodies (Figure 3K). These results suggest that HIF‐1α increases the synthesis of PC in CM cells through the triglyceride–diacylglycerol–PC pathway.

**FIGURE 4:**
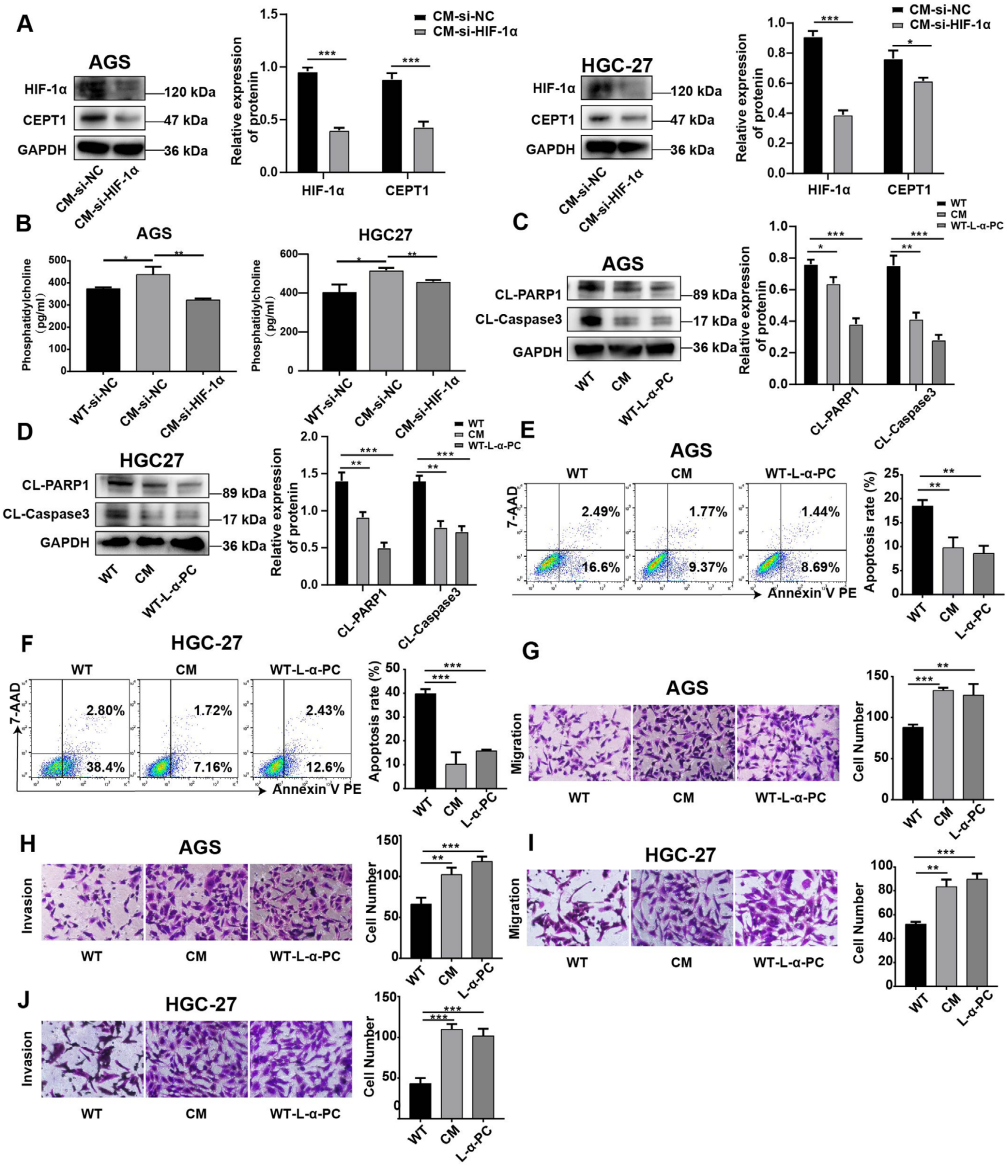
HIF‐1α promotes the metastatic and antiapoptotic capacities of CM cells by regulating PC metabolism. (A) Western blot analysis of CEPT1 protein levels in HIF‐1α‐knockdown CM from GC cells. (B) The PC content in the supernatants of CM‐AGS and CM‐HGC27 with HIF‐1α knockdown was detected via ELISA. (C, D) The protein levels of HIF‐1α, CL‐PARP1 and CL‐Caspase3 in AGS and HGC27 following treatment with L‐α‐PC were analysed via Western blotting. (E, F) The apoptosis rates of the WT, CM and WT‐L‐α‐PC group were detected by flow cytometry. (G–J) The migration and invasion of the WT, CM and WT‐L‐α‐PC groups were assessed using the Transwell method. The experiment was performed in three separate sessions. The values are expressed as the standard deviation (SD), and Student's t test was used to assess statistical significance. Nonsignificant results are represented by ‘ns’, and significant levels are represented by *p < 0.05, **p < 0.01 and ***p < 0.001.

Next, we asked whether HIF‐1α modulates the triglyceride–diacylglycerol–PC pathway in CM GC cells. Western blot analysis revealed that HIF‐1α knockdown significantly reduced the protein level of CEPT1 in CM‐GC cells (Figure 4A). In addition, we observed that the PC content in the supernatants of CM‐AGS and CM‐HGC27 cells was inhibited by HIF‐1α knockdown (Figure 4B). Moreover, we observed that treatment with L‐α‐phosphatidylcholine (L‐α‐PC), a natural PC, markedly decreased the apoptosis of GC cells under hypoxic conditions (Figure 4C–F) and enhanced the migration and invasion of GC cells (Figure 4G–J), similar to CM GC cells. These results suggest that the HIF‐1α‐induced metastatic and antiapoptotic capacities of the CM GC cells may be mediated by PC metabolism.

### 3.4 CM Cells Promote Lung Metastasis in Mice by Partially Regulating the HIF‐1α/CEPT1/PC Axis

**FIGURE 5:**
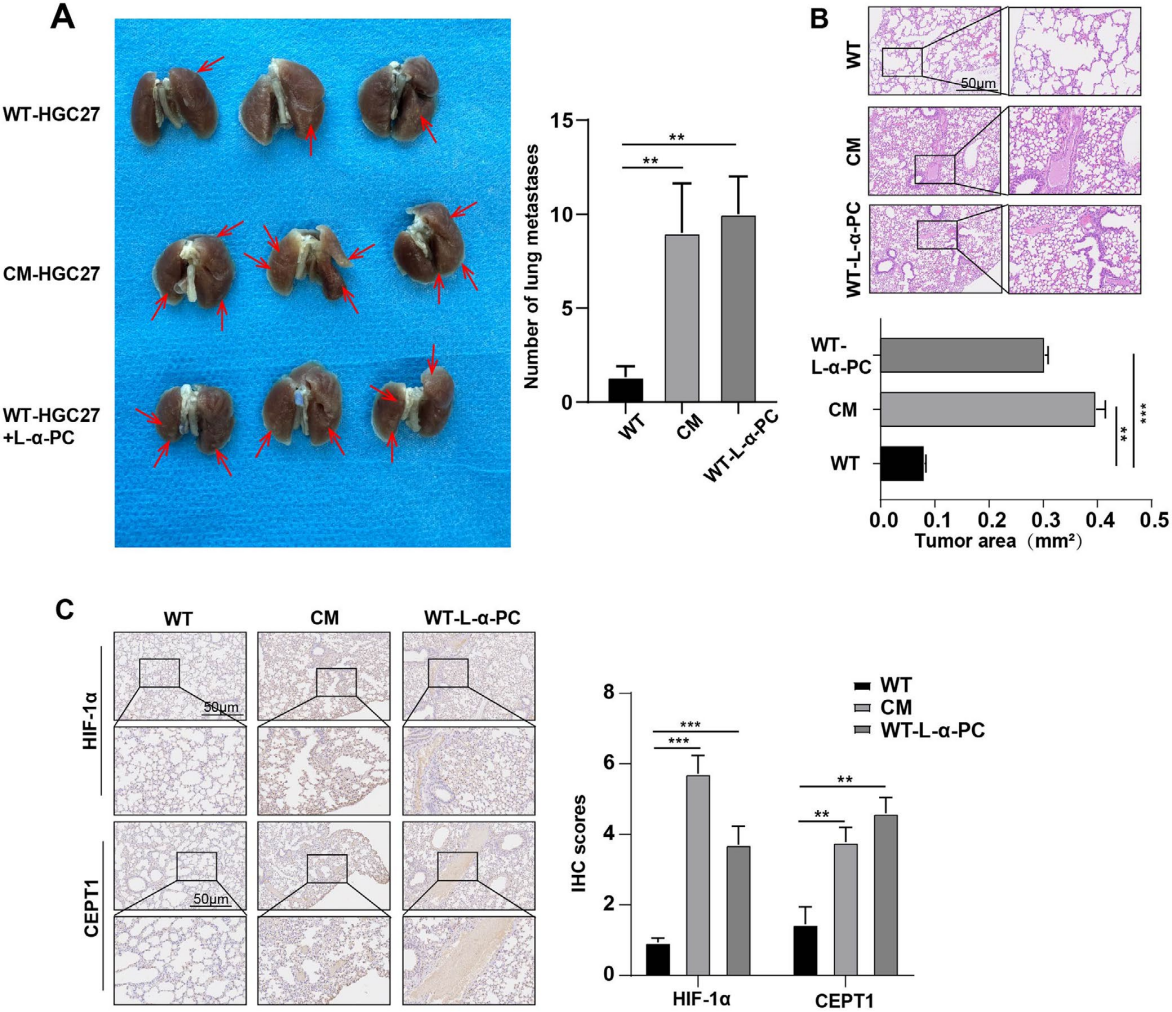
CM cells promote lung metastasis in mice via the HIF‐1α/CEPT1/PC axis. (A) Lung images of the mice in the HGC27, CM‐HGC27 and L‐α‐PC groups. (B) Typical HE staining images of lung metastases from the WT, CM and WT‐L‐α‐PC group. Scale, 50 μm. (C) The expression of HIF‐1α and CHPT1 in the lung tissues of the mice in the WT, CM and WT‐L‐α‐PC groups was detected via immunohistochemistry. Scale, 50 μm. Significant levels are represented by **p < 0.01 and ***p < 0.001.

To assess the potential of CM cells to promote lung metastasis in mice via the HIF‐1α/CEPT1/PC axis, we established a murine model of lung metastasis by intravenously injecting WT or CM HGC27 cells via the tail vein. As shown in Figure 5A,B, the number of metastatic nodules in the lung tissue of the mice in the CM‐HGC27 and WT + L‐α‐PC groups was significantly greater than that in the WT group. In addition, the IHC results revealed that the expression of HIF‐1α and CEPT1 in the CM‐HGC27 and WT + L‐α‐PC groups was greater than that in the WT group (Figure 5C). These results suggest that CM GC cells are prone to metastasis in vivo, which is partially associated with the HIF‐1α/CEPT1/PC axis.

### 3.5 High HIF‐1α and CEPT1 Expression Is Associated With Poor Prognosis in GC Patients

**FIGURE 6:**
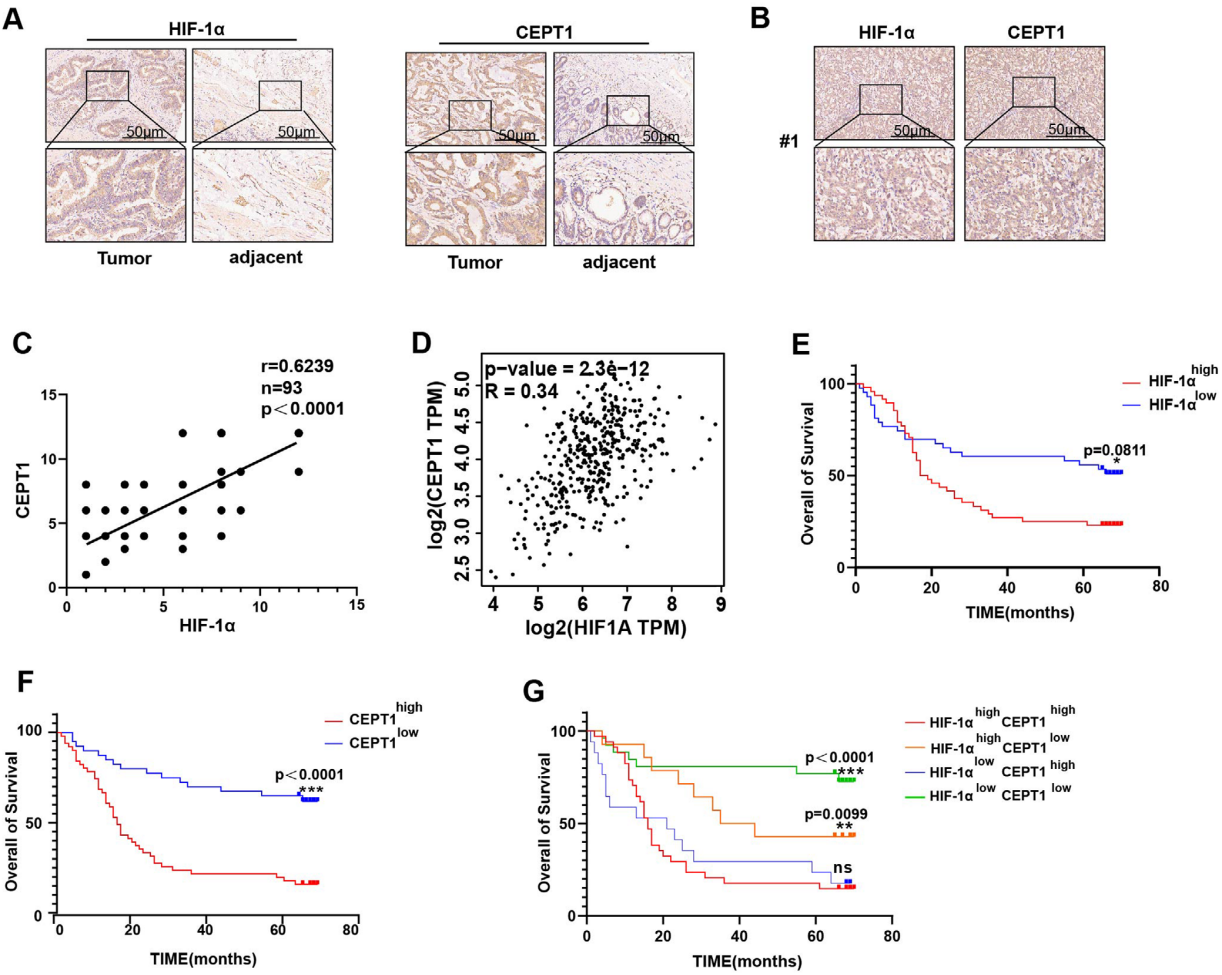
High expression of HIF‐1α and CEPT1 is a poor prognostic factor for gastric cancer. (A) Typical images of HIF‐1α and CEPT1 immunohistochemical staining of tumour and adjacent tissue from clinical specimens of GC patients. Scale, 50 μm. (B) Immunohistochemical staining of HIF‐1α and CEPT1 in #1 GC patient tissue. Scale, 50 μm. (C) Correlation analysis of HIF‐1α and CEPT1 expression in GC tissues. (D) Correlation analysis of HIF‐1α and CEPT1 expression in gastric cancer tissues using the GEPIA database. (E, F) Kaplan–Meier curves of HIF‐1α and CEPT1 in patients with GC. (G) Kaplan–Meier curves of the overall survival of patients with GC stratified according to HIF‐1α and CEPT1 expression were generated and divided into four subgroups. Nonsignificant results are represented by ‘ns’, and significant levels are represented by *p < 0.05, **p < 0.01 and ***p < 0.001.

As shown in Figure 6A, the expression of HIF‐1α and CEPT1 in GC tumour tissues was significantly greater than that in adjacent tissues. Moreover, a correlation analysis revealed a significant positive correlation between HIF‐1α and CEPT1 expression in the cancer tissues of GC patients (Figure 6B,C). In addition, this positive correlation was also observed in gastric cancer tissues from the GEPIA database (http://gepia.cancer‐pku.cn/) (Figure 6D). Moreover, according to the clinical indicators of 93 GC patients, the expression levels of HIF‐1α and CEPT1 were closely related to N stage (Table S2). Kaplan–Meier survival analysis revealed that high HIF‐1α expression was associated with a worse prognosis (hazard ratio: 1.936, 95% CI: 1.142–3.281), and the same result was obtained in the group with high CEPT1 expression (hazard ratio: 3.670, 95% CI: 2.151–6.261) (Figure 6E,F). Importantly, the prognosis of GC patients with high expression of both HIF‐1α and CEPT1 was significantly worse than that of patients in the other three groups (Figure 6G). Thus, we suggest that increased expression of HIF‐1α and CEPT1 is positively correlated in GC specimens and predicts poor prognosis.

### 3.6 CM Increases the Stability of HIF‐1α Through the Ubiquitin‐Proteasome System

**FIGURE 7:**
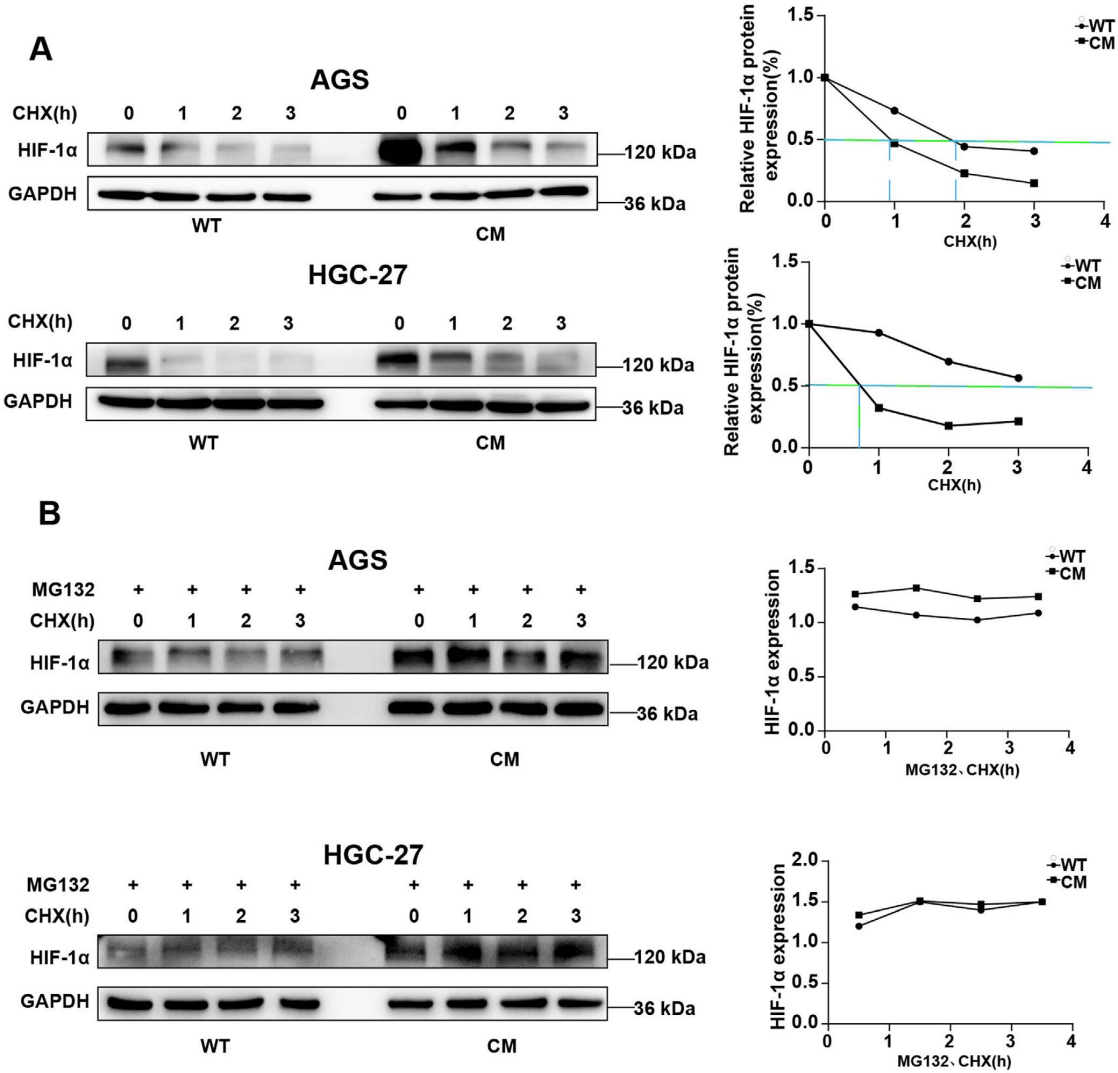
CM enhances the stability of HIF‐1α in GC cells through the ubiquitin‐proteasome system. (A) Western blot analysis was used to detect changes in HIF‐1α protein levels in CM‐AGS cells and CM‐HGC27 cells treated with actinomycosterone (CHX) for 1, 2 and 3 h. (B) Changes in HIF‐1α protein levels in CM‐AGS cells and CM‐HGC27 cells treated with a proteasome inhibitor (MG132) for 1, 2 and 3 h were detected. The experiment was performed in three separate sessions. The values are expressed as the standard deviation (SD), and Student's t test was used to assess statistical significance. Nonsignificant results are represented by ‘ns’, and significant levels are represented by *p < 0.05, **p < 0.01 and ***p < 0.001.

Next, we investigated the cause of high HIF‐1α expression in CM GC cells. Given that there was no difference in *HIF‐1α* mRNA expression between WT and CM GC cells (Figure 2A), we hypothesised that HIF‐1α protein expression is modulated at the posttranscriptional level in CM GC cells. To test this hypothesis, cycloheximide (CHX), a protein synthesis inhibitor, was used to block the protein synthesis of HIF‐1α, after which the degradation rate of the HIF‐1α protein in the CM group was significantly lower than that in the WT group (Figure 7A). Previous studies have shown that HIF‐1α degradation is closely associated with the proteasome pathway [21, 22]. Therefore, we used the proteasome inhibitor MG132 to verify whether HIF‐1α was degraded in CM GC cells via the proteasome pathway. As shown in Figure 7B, the degradation of HIF‐1α protein in both WT and CM GC cells was blocked by MG‐132 treatment. These findings suggest that CM enhances the stability of HIF‐1α in GC cells through the ubiquitin‐proteasome system.

## 4 Discussion

It is widely known that CM is a crucial process in the metastatic cascade of cancer cells and that preventing the migration of cancer cells in confined spaces can halt tumour metastasis [5]. However, the molecular mechanisms underlying CM in GC remain unclear. In this study, we constructed a CM GC cell model to imitate the confined microenvironment conducive to the metastasis of GC. We demonstrated that CM cells exhibited heightened metastatic and antiapoptotic capacities, which is consistent with previous reports [23, 24]. Moreover, we observed that the protein levels of HIF‐1α were markedly increased in CM GC cells, suggesting that HIF‐1α may play important roles in CM GC cells.

As a key signal transduction molecule activated under hypoxic conditions, HIF‐1α expression is generally elevated in tumours and performs crucial biological functions, including metastasis, in cancers [25]. For example, SUMOylation‐dependent HIF‐1α/CLDN6 negative feedback contributes to the metastasis of breast cancer [26]. Xia et al. reported that hypoxic GC‐derived exosomes carrying miR‐301a‐3p enhanced HIF‐1α accumulation, promoting the malignant behaviour and metastasis of GC [27]. In this study, we found that HIF‐1α was highly expressed in the cancer tissues of GC patients and was associated with N stage. Furthermore, increased HIF‐1α expression predicted poor prognosis. Importantly, the silencing of HIF‐1α counteracted the effect of CM on the antiapoptotic and metastatic ability of GC cells, suggesting crucial roles of HIF‐1α in CM from patients with GC.

HIF‐1α has been demonstrated to increase the malignant transformation potential of various tumours through the regulation of tumour metabolic reprogramming [28]. Therefore, we investigated whether HIF‐1α modulated the metastatic and antiapoptotic capacities of CM GC cells via metabolic reprogramming. The results of the nontargeted metabolomics analysis using WT and CM GC cells revealed that PC was markedly upregulated in CM cells. While we did not perform non‐targeted metabolomics on AGS cells, our findings indicate that HIF‐1α enhances the metastatic and anti‐apoptotic capacities of both CM HGC27 and AGS cells through PC metabolism. PC plays a crucial role in the composition of eukaryotic cell membranes and serves as a signalling molecule involved in the regulation of cell proliferation and apoptosis [29]. Previous research indicates that tumours often exhibit reprogrammed lipid synthesis and metabolism to provide the necessary survival materials and growth energy, thereby satisfying the requirements for the malignant growth and metastasis of tumour cells [30]. The synthesis of PC consists of two pathways: the Kennedy pathway and the triglyceride–diacylglycerol–PC pathway [19, 20]. In this study, we found that the raw material choline and the key enzyme CHPT1 of the Kennedy pathway were not significantly different in CM GC cells. In contrast, the contents of triglycerides and diacylglycerol, two raw materials of the triglyceride–diacylglycerol–PC pathway, and the key enzyme CEPT1 were significantly increased in CM GC cells. Moreover, there was a significant positive correlation between HIF‐1α and CEPT1 expression in the cancer tissues of GC patients. GC patients with high expression of both HIF‐1α and CEPT1 had the worst prognosis. Importantly, HIF‐1α knockdown markedly suppressed CEPT1 expression and PC levels in CM GC cells. In addition, treatment with L‐α‐PC, a natural PC, markedly decreased the apoptosis of GC cells under hypoxic conditions and enhanced the migration and invasion of GC cells. Importantly, ChIP analysis showed significant enrichment of DNA precipitated by anti‐HIF‐1α antibody in the CEPT1 promoter sequence. Our findings indicate that the HIF‐1α‐induced metastatic and antiapoptotic capacities of CM GC cells are mediated through triglyceride–diacylglycerol–PC metabolism. However, the precise mechanism by which HIF‐1α promotes triglyceride–diacylglycerol–PC metabolism in CM GC cells is still unclear. Therefore, it will be important to further explore the molecular mechanism by which HIF‐1α modulates triglyceride–diacylglycerol–PC metabolism in CM GC cells in the future. Although the impact of HIF‐1α on PC metabolism in CM cells is important, other mechanisms of HIF‐1α‐mediated regulation of CM cannot be disregarded. For instance, our nontargeted metabolomics data demonstrated that the levels of phosphatidylethanolamine (PE) were also increased in CM cells compared with WT cells, implying that HIF‐1α may modulate the function of CM cells via PE. It may be valuable to explore whether HIF‐1α modulates CM of GC cells via the PE.

Previous studies have demonstrated that HIF‐1α expression is regulated by posttranscriptional mechanisms, including the ubiquitin‐proteasome pathway [31, 32]. A study conducted on human cancers overexpressing NAD(P)H:quinone oxidoreductase 1 (NQO1) revealed that NQO1 binds directly to the HIF‐1 protein, inhibiting PDH‐mediated proteasomal degradation and stabilising HIF‐1 [21]. Liu et al. noted that 14‐3‐3σ enhances the binding of NEDD4L to HIF‐1α, leading to an increase in HIF‐1α polyubiquitination and promoting its degradation via the proteasome [33]. In this study, we observed that there was no statistically significant difference in the expression of HIF‐1α mRNA between CM cells and WT cells; the HIF‐1α protein levels were significantly increased in CM cells. This suggests that the expression of HIF‐1α protein in CM GC cells is primarily regulated at the posttranscriptional level, rather than at the transcriptional level. Then, we demonstrated that high protein expression of HIF‐1α was regulated in CM GC cells via the ubiquitin‐proteasome pathway. However, we did not explore the molecular mechanisms by which the ubiquitin‐proteasome pathway modulates the expression of HIF‐1α in CM GC cells. Therefore, further investigation is still needed. In addition, HIF‐1α is also modulated by other posttranscriptional mechanisms, such as acetylation and phosphorylation [34, 35]. Therefore, we cannot exclude the possibility that HIF‐1α is regulated in CM GC cells through acetylation or phosphorylation. HIF‐1α inhibitors, evaluated both as monotherapy and in combination with other agents, have shown potential in treating advanced or refractory cancers [36]. For instance, 2ME2 NCD (Panzem), an endogenous estradiol metabolite that suppresses HIF‐1α protein synthesis and transcriptional activity, has been studied in a Phase II trial. In this trial, 18 patients with relapsed platinum‐refractory ovarian cancer, who had undergone a median of five prior treatments, were enrolled. While the primary endpoint of objective response rate (ORR) was not met (ORR = 0), 7 patients (31.3%) demonstrated stable disease, and the treatment exhibited a favourable tolerability profile [37]. These findings indicate that targeting or modulating HIF‐1α may hold significant therapeutic potential.

However, the ‘drug‐ability’ of HIF‐1α as an oncogenic transcription factor still faces huge challenges. Karamouzis et al. believe that gene regulation is mainly achieved at the transcriptional level, and transcription factors play a key role in this process [38]. Based on the analysis of the commonality of transcription factor targeting by Karamouzis et al., we can directly target the binding of HIF‐1α to the P3 site in the promoter region of its target gene CEPT1 by designing competitive inhibitors. However, competitive inhibitors may fail to achieve efficient blocking due to insufficient binding energy or spatial locus obstruction. Small interfering RNAs can silence the expression of the oncogene HIF‐1α and thereby inhibit tumour growth. However, inhibiting HIF‐1α may interfere with the hypoxia adaptation of normal tissues, making the trade‐off of the treatment window particularly important. Furthermore, a single inhibition of the HIF‐1α/CEPT1/PC signalling pathway may lead to the activation of the compensatory pathway. In the future, the integration of interdisciplinary technologies may help develop anti‐cancer therapeutic drugs targeting transcription factors with high selectivity and low toxicity potential.

In conclusion, our study investigated the effect of HIF‐1α on the CM of GC cells. Our findings indicated that HIF‐1α promotes the metastatic and antiapoptotic capacities of CM GC cells by partially regulating the CEPT1/PC axis. Therefore, blockade of the HIF‐1α/CEPT1/PC signalling axis has emerged as a promising therapeutic strategy for preventing cancer cell metastasis in GC.

## Author Contributions

**Ming Zhou:** writing – original draft. **Kanger Shen:** visualisation. **Ziyi Huang:** visualisation. **Qin Zhan:** data curation. **Jiayu Wang:** software. **Xiaozhe Mao:** visualisation. **Tongguo Shi:** writing – review and editing. **Rui Li:** writing – review and editing.

## Ethics Statement

The experiments on clinical gastric cancer samples were approved by the Medical Ethics Committee of the First Affiliated Hospital of Soochow University (Ethics No. 2024541). Animal experiments were approved by the Animal Ethics Committee of Soochow University (Suzhou, China, Ethics No. 202408A0217).

## Conflicts of Interest

The authors declare no conflicts of interest.

## Supporting information


**Figure S1.** The Western blot band densities of CHPT1 and CEPT1 in Figure 3I were quantified using ImageJ software.

**Table S1.** Non‐targeted metabolomics analysis of the abundance of differential metabolites in WT‐HGC27 and CM‐HGC27 cells.

**Table S2.** Expression and clinical features of HIF‐1α and CEPT1 in patients with GC.

## Data Availability

The data that support the findings of this study are available from the corresponding author upon reasonable request.
