# Supplementary material for: HIF‐1α Promotes the Confined Migration of Gastric Cancer Cells by Modulating Phosphatidylcholine Metabolism
Source: J Cell Mol Med. 2025 Sep 15;29(18):e70828. doi: 10.1111/jcmm.70828 (PMC12436174; doi:10.1111/jcmm.70828)
Supplement: Supplementary file 1 — Figure S1. The Western blot band densities of CHPT1 and CEPT1 in Figure 3I were quantified using ImageJ software. [file JCMM-29-e70828-s002.docx]

Supplementary figures 1

**Figure S1.** The Western blot band densities of CHPT1 and CEPT1 in Figure 3I were quantified using ImageJ software. Nonsignificant results are represented by "ns", and significant levels are represented by ***p< 0.001.

**
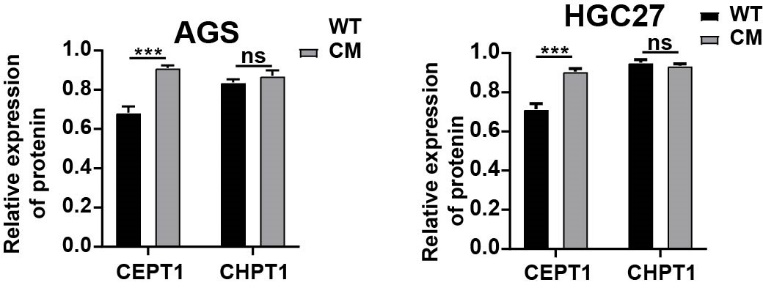
**
