# Supplementary material for: HIF‐1α Promotes the Confined Migration of Gastric Cancer Cells by Modulating Phosphatidylcholine Metabolism
Source: J Cell Mol Med. 2025 Sep 15;29(18):e70828. doi: 10.1111/jcmm.70828 (PMC12436174; doi:10.1111/jcmm.70828)
Supplement: Supplementary file 3 — Table S2. Expression and clinical features of HIF‐1α and CEPT1 in patients with GC. [file JCMM-29-e70828-s003.docx]

**Supplementary table 2：Expression and clinical features of HIF-1α and CEPT1 in patients with GC**

| **Characteristic** | **HIF-1α expression** | | ***P*** | **CEPT1 expression** | | ***P*** |
| --- | --- | --- | --- | --- | --- | --- |
|  | **Low** | **High** |  | **Low** | **High** |  |
| Gender  Male  Female | 26  17 | 33  17 | 0.386 | 23  17 | 36  17 | 0.207 |
| Age  ＜=60  ＞60 | 30  13 | 36  14 | 0.496 | 27  13 | 39  14 | 0.340 |
| T  T1-2  T3-4 | 7  35 | 8  41 | 0.593 | 9  30 | 6  46 | 0.119 |
| N  N0  N1-3 | 14  29 | 0  41 | 1.9986e^-7^* | 16  24 | 6  47 | 0.001* |
| M  M0  M1 | 42  1 | 50  0 | 0.462 | 40  0 | 52  1 | 0.570 |
| AJCC stage  I-II  III-IV | 7  36 | 14  36 | 0.136 | 8  32 | 13  40 | 0.398 |
